# Combined therapy of CAR-IL-15/IL-15Rα-T cells and GLIPR1 knockdown in cancer cells enhanced anti-tumor effect against gastric cancer

**DOI:** 10.1186/s12967-024-04982-6

**Published:** 2024-02-18

**Authors:** Jianbin Ye, Qiaoyuan Liu, Yunxuan He, Zhenkun Song, Bao Lin, Zhiwei Hu, Juanyuan Hu, Yunshan Ning, Cheguo Cai, Yan Li

**Affiliations:** 1 School of Laboratory Medicine and Biotechnology, Southern Medical University, Guangzhou, 510515 Guangdong People’s Republic of China; 2 Shenzhen Beike Biotechnology Co., Ltd., Shenzhen, 518000 Guangdong People’s Republic of China

**Keywords:** CAR-T, Solid tumor, Gastric cancer, IL-15/IL-15Rα, GLIPR1, Combined therapy

## Abstract

**Background:**

Chimeric antigen receptor (CAR) T cell therapy has shown remarkable responses in hematological malignancies with several approved products, but not in solid tumors. Patients suffer from limited response and tumor relapse due to low efficacy of CAR-T cells in the complicated and immunosuppressive tumor microenvironment. This clinical challenge has called for better CAR designs and combined strategies to improve CAR-T cell therapy against tumor changes.

**Methods:**

In this study, IL-15/IL-15Rα was inserted into the extracellular region of CAR targeting mesothelin. In-vitro cytotoxicity and cytokine production were detected by bioluminescence-based killing and ELISA respectively. In-vivo xenograft mice model was used to evaluate the anti-tumor effect of CAR-T cells. RNA-sequencing and online database analysis were used to identify new targets in residual gastric cancer cells after cytotoxicity assay. CAR-T cell functions were detected in vitro and in vivo after GLI Pathogenesis Related 1 (GLIPR1) knockdown in gastric cancer cells. Cell proliferation and migration of gastric cancer cells were detected by CCK-8 and scratch assay respectively after GLIPR1 were overexpressed or down-regulated.

**Results:**

CAR-T cells constructed with IL-15/IL-15Rα (CAR-ss-T) showed significantly improved CAR-T cell expansion, cytokine production and cytotoxicity, and resulted in superior tumor control compared to conventional CAR-T cells in gastric cancer. GLIPR1 was up-regulated after CAR-T treatment and survival was decreased in gastric cancer patients with high GLIPR1 expression. Overexpression of GLIPR1 inhibited cytotoxicity of conventional CAR-T but not CAR-ss-T cells. CAR-T treatment combined with GLIPR1 knockdown increased anti-tumor efficacy in vitro and in vivo.

**Conclusions:**

Our data demonstrated for the first time that this CAR structure design combined with GLIPR1 knockdown in gastric cancer improved CAR-T cell-mediated anti-tumor response.

**Supplementary Information:**

The online version contains supplementary material available at 10.1186/s12967-024-04982-6.

## Background

Since the approvals of two CD19-targeting CAR-T (Kymriah and Yescarta) by FDA in August 2017, the chimeric antigen receptor T (CAR-T) cell therapy has achieved tremendous success in hematological cancers [1, 2]. However, CAR-T cell therapy on solid tumors is less successful with several obstacles: lack of homing and trafficking, suboptimal persistence, immunosuppressive tumor microenvironment (TME), antigen escape and heterogeneity [3]. To address these problems, innovative T cell engineering strategies have been developed to modify the CAR structures [4, 5]. Briefly, incorporation of costimulatory signaling domains can improve the proliferation, persistence, and cytotoxicity of CAR-T cells [6], while transcribing and secreting an immune modifier into the extracellular fluid can improve CAR-T cells resistance to TME-induced effects and activate the immune system [7]. Another way for CAR-T improvement is to combine immunogenic chemotherapy and/or checkpoint blockade with CAR-T therapy [8, 9]. For example, combined administration of cyclophosphamide and oxaliplatin can activate tumor macrophages to express chemokines, which recruited CAR-T cells, improved CAR-T cell infiltration and increased tumor sensitivity to anti-PD-L1 in lung adenocarcinoma [9]. But to construct an effective CAR and to find suitable targets for combined therapies in different cancers are still with many difficulties.

IL-15 superagonist (combination of IL-15 with IL-15 receptor alpha in solution) shows advantages over monomeric IL-15 with prolonged half-life and more potently stimulating natural killer cells (NK cells) and CD8^+^ T cells [10]. Genetically engineered cells co-expressing IL-15/IL-15Rα complex for secretion is also emerging for cancer treatment [11]. All forms have demonstrated efficacy in causing tumor regression in animal experiments [12, 13], which provides strong rationale for advancing IL-15 superagonist through clinical trials [14, 15]. Thus, inserting the IL-15/IL-15Rα into the CAR structure is an alternative way to enhance CAR-T cell function.

Through phenotypic changes, cancer cells can escape from immune surveillance and elimination by immune effector cells. Previous studies based on cancer patients treated with immunotherapy have provided several mechanisms that promote immune evasion, such as defects in antigen presentation and in interferon-receptor signaling [16–18]. But these studies are limited to the detection of frequently occurring genetic alterations. Lawson, K.A., et al. unveiled a set of genes and pathways of cancer cells that phenotypically facilitate cancer-intrinsic cytotoxic T lymphocytes (CTLs) evasion in mouse cancer models [19]. However, much remained to be revealed in human cancer models and under different cancer types. Targeting new mechanism of tumor resistance to CTLs is a feasible way for combined therapy.

In this study, we constructed a new CAR structure with an IL-15/IL-15Rα sushi domain (CAR-ss-T) on the CAR extracellular region, which facilitate cytotoxicity against gastric cancer cells compared with conventional CAR. RNA sequencing of the remaining cancer cells after low-efficiency target cell killing revealed that GLI Pathogenesis Related 1 (GLIPR1) was up-regulated and high expression of GLIPR1 decreased survival rate of stomach adenocarcinoma (STAD) patients. Knockdown of GLIPR1 enhanced CAR-T cells function. This research provided a more effective CAR structure for cancer and potential therapeutic targets for combined immune therapies with CAR-T cells.

## Methods

### *In-vitro* culture and lentivirus transduction of human T cell

Peripheral blood mononuclear cells (PBMC) from healthy donors were purchased from Milestone Biotechnologies (Shanghai, China) and handled with necessary safety procedures and ethical requirements. The PBMC were activated by T cell TransAct (130-111-160, Miltenyi Biotec, USA) according to the manufacturer’s instructions and cultured in RPMI1640 containing 10% fetal bovine serum (Gibco, USA), and 200 U/ml human IL-2 (S10970015, Beijing Four Rings Bio-Pharmaceutical, China). After 72 h, lentiviral particles were added to the cultures at a multiplicity of infection (MOI) of 10. After transduction for 24 h, the CAR-T cells were cultured in fresh medium to maintain the cell density at 1 × 10^6^ cells/ml. The culture medium was replenished every 2 days.

### Cytotoxicity assays

The target cancer cells were incubated with CAR-T cells at the ratio of 1:5 and 1:2 in sextuplicate wells of 96-well plates. After indicated time, target cell viability was monitored by adding 100 µl/well of the substrate D-luciferin (potassium salt) (ab143655, Abcam, USA) at 150 µg/ml. Background luminescence was negligible (< 1% of the signal from wells containing only target cells). The viability (%) was calculated as experimental signal/maximal signal × 100, and the percent lysis was equal to 100% viability.

### Flow cytometry assay

Flow cytometry was performed on a BD LSRFortessa cytometer, and data were analyzed using FlowJo software. The antibodies for flow cytometry include Alexa Fluor® 647 Anti-Mesothelin antibody (252135, Abcam, USA), Alexa Fluor® 647 Rabbit IgG (199093, Abcam, UK), PE-Labeled Human Mesothelin (MSN-HP2H5, Acrobiosystems, China), and APC-anti human CD215 (530203, Biolegend, USA).

### Cytokine secretion assay

The effector cells (15B6 CAR-T and 15B6ss CAR-T cells) were co-cultured with target cells for 24 h at an E:T ratio of 5:1, 2:1 and 1:1, and the medium supernatant was assessed for the levels of cytokine secretion. The concentration of IL-2, TNF-α, IFN-γ and granzyme B was measured using enzyme-linked immunosorbent assay kits (Biolegend, USA) according to the manufacturer’s instructions.

### Xenograft mouse model

NOD-Prkdcscid IL2rgem1/Cyagen (C-NKG) mice were purchased from Jiangsu Cyagen Biosciences Co., Ltd. (Shanghai, China). Six to eight-week-old C-NKG mice were bred under specific pathogen-free conditions. Cancer cells (at a density of 1 × 10^8^ cells/ml) were suspended in saline and subcutaneously injected into C-NKG mice with 50 μl at right flank. After 7 days, the mice were anaesthetized and imaged using IVIS system followed by intraperitoneal injection of 150 mg/kg D-luciferin (Abcam, USA). When the mean tumor volume reached ~ 50 mm^3^ (Volume = Length × Width^2^/2), mice were treated with 100 μl of untransduced T cells, 15B6 CAR-T, or 15B6ss CAR-T cells (2 × 10^7^ cells/ml or 5 × 10^7^ cells/ml) by intravenous injection. The bioluminescent signals, tumor volume and body weight were measured every four days. The data were quantified using Living Image software (Caliper Life Science, USA). The mice were euthanized when the tumor volume reached 2000 mm^3^. All animal protocols were approved by the Institutional Human Ethics Review Board of Southern Medical University, China.

### Cancer cells and T cell line culture

ASPC1, HGC27, MKN45, NCI-H520, Hut78 and Jurkat cells were purchased from Nanjing Cobioer Biosciences (Nanjing, China). The cells were tested via short tandem repeat (STR) profiling. ASPC1, HGC27, MKN45, NCI-H520 and Jurkat cells were cultured in RPMI1640 (22400-089, Gibco, USA) containing 10% fetal bovine serum (10091-148, Gibco, USA). ASPC1-luc, HGC27-luc, MKN45-luc, and NCI-H520-luc were constructed by lentivirus infection (H7656, OBiO Technology, China) and cultured in RPMI1640 containing 10% fetal bovine serum, and 2 μg/ml Puromycin (A11138-02, Gibco, USA). Hut78 was cultured in IMDM containing 10% fetal bovine serum. All cell lines were cultured in a humidified incubator with 5% CO2 at 37 °C.

### CAR construction and lentivirus production

The MSLN-targeting scFv 15B6 [20] and the MSLN-targeting VHH C4 were synthesized (IGEbio, China) and cloned into two CAR-encoding lentivirus backbones respectively and named 15B6 CAR, 15B6ss CAR, C4 CAR and C4ss CAR. 15B6 CAR and C4 CAR contain a CD8 hinge spacer and transmembrane domain, CD28, 4-1BB, and CD3ζ orderly. 15B6ss CAR and C4ss CAR contain an IL-15Rα domain, CD8 hinge spacer and transmembrane domain, CD28, 4-1BB, CD3ζ and IL-15 orderly. The CARs were cloned into the pRRLSIN.cPPT-GFP.WPRE vector (Addgene, USA) and expressed under the control of an EF1α promoter. Human embryonic kidney 293 T cells were seeded at T175 flask and cultured in DMEM (22400-089, Gibco, USA) containing 10% fetal bovine serum (10091-148, Gibco, USA). The 293 T cells were co-transfected with lentiviral vector plasmid including the recombinant expression vector DNA, lentiviral packaging plasmid pMDLg/pRRE, pRSV-Rev and pMD2.G (Addgene, USA) using polyethylenimine hydrochloride (24765-1, Polysciences, USA). The viral supernatants were harvested every 24 h for three times after transfection and the lentiviral particles were concentrated 100-fold by PEG6000 (528877-1 KG, Millipore, USA) for 15 min at 8000 g.

### CAR-T cell proliferation assay

To analyze the proliferation of CAR-T cells, cell numbers were counted for 3 days after virus transduction. T cells were dyed by AOPI (CS2-0106-5 ml, Nexcelom, USA) and counted by Cellometer Auto2000 (Nexcelom, USA).

### Real-time PCR for gene expression analysis

RNA was extracted by RNA Easy Fast Tissue/Cell Kit (DP451, TIANGEN, China). The first-strand cDNA was then synthesized with PrimeScript™ RT reagent Kit with gDNA Eraser (RR047A, Takara, Japan). Quantitative real-time PCR was performed in LightCycler 480 II (Roche, Switzerland) using TB Green® Premix Ex Taq™ II FAST qPCR (CN830A, Takara, Japan). The relative mRNA expression level was calculated by the threshold cycle (Ct) value of each PCR product and normalized with β-actin by using a comparative 2^−ΔΔCt^ method.

### Tumor cell proliferation assay

To analyze the proliferation of cancer cells, cells were seeded into 96-well plate with 5000 cells per well in 0.1 ml medium and 6 repeats per group. Three plates were seeded for the detection after 24 h, 48 h and 72 h respectively by Cell Counting Kit-8 (E606335-2000, Sangon Biotech, China).

### Scratch assay

To examine cancer cell migration, cells were seeded in 6-well plate and cultured until confluence. Cells were scraped with a 200 μl micropipette tip and monitored at 0 h and 12 h. The uncovered wound area was measured and quantified by OLYMPUS cellSens Dimension 2.1 (OLYMPUS, Japan).

### Online database data analysis

Gene Expression Profiling Interactive Analysis (GEPIA2, http://gepia2.cancer-pku.cn/#index) was developed based on the The Cancer Genome Atlas Program (TCGA) and Genotype-Tissue Expression (GTEx) databases. The GEPIA2 database was used to analyze expression of GLIPR1 in STAD patients and normal person and survival analysis in STAD patients. The University of ALabama at Birmingham CANcer data analysis Portal (UALCAN, https://ualcan.path.uab.edu/analysis.html) was developed based on the TCGA dataset. The UALCAN was used to analyze gene expression of GLIPR1 in STAD patients based on tumor grades.

### RNA-sequencing and data analysis

The effector cells (15B6 CAR-T cells) were co-cultured with target cells (HGC27 and MKN45) for 24 h under E:T ratio of 1:1, then the medium supernatant was removed and the remained cancer cells were washed by PBS for three times. The remained cancer cell samples were lysed by RNAiso Plus (9108, Takara, Japan) and sent to IGEbio (Guangzhou, China) for bulk-cell RNA-sequencing and data analysis.

### Statistics analysis

The data were presented as the mean ± standard error of the means. Independent-samples T test (two-tailed) and one-way ANOVA were used to determine the statistical significance of differences between samples. ^*^p < 0.05 and ^**^p < 0.01 indicated statistical significance. All statistical analyses were performed using IBM SPSS statistics 20 (IBM corporation, USA).

## Results

### CAR-ss-T cells increased cell viability, cytokines production and cytotoxicity against gastric cancer compared with conventional CAR-T

**Fig. 1:**
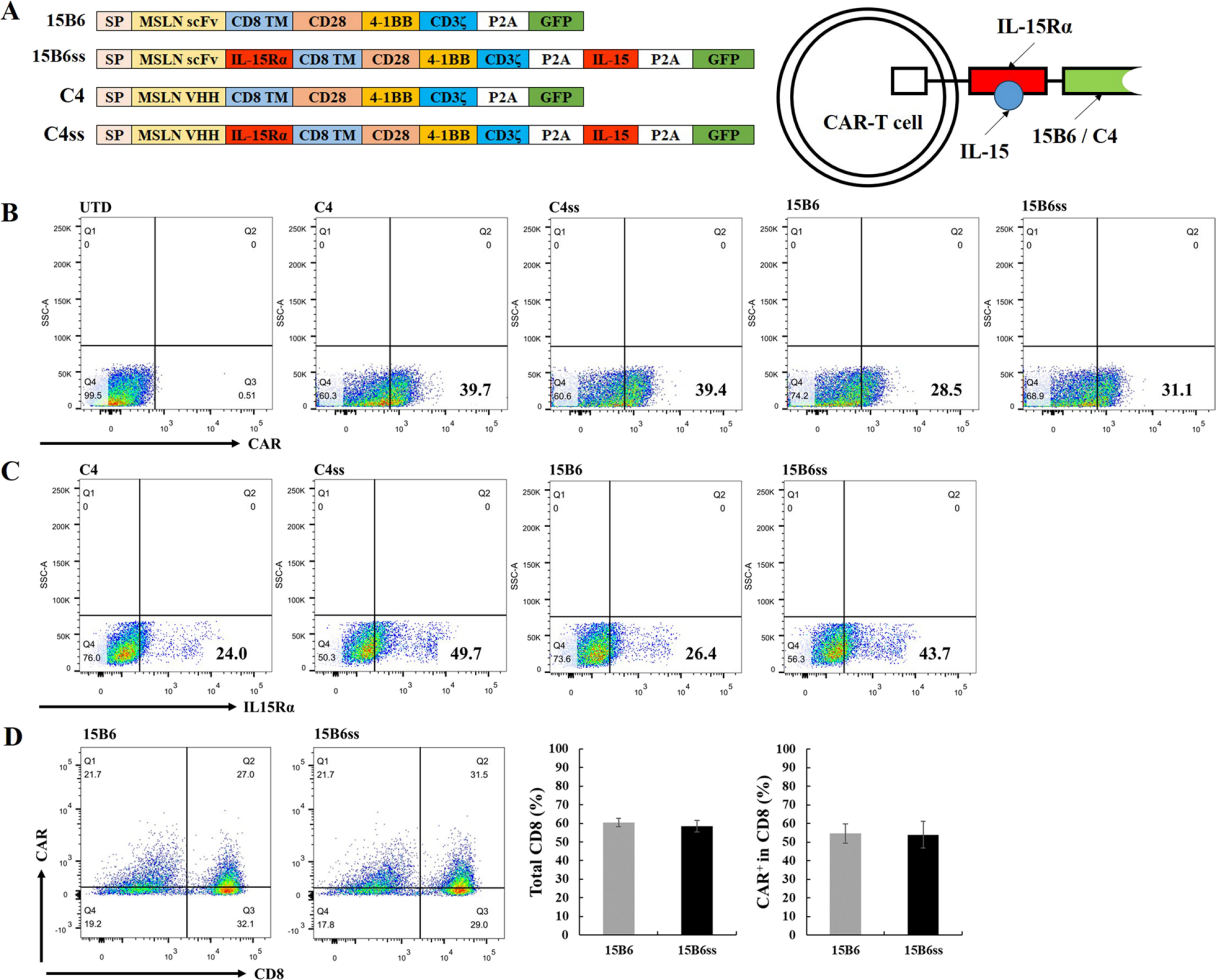
Construction of MSLN-specific CAR-IL-15/IL-15Rα-T cells. **A** Construction of CAR with IL-15/IL-15Rα. SP represented signal peptide. TM represented transmembrane domain. 15B6 represented CAR constructed with anti-MSLN scFv. 15B6ss represented CAR constructed with anti-MSLN scFv and IL-15/IL-15Rα. C4 represented CAR constructed with anti-MSLN VHH. C4ss represented CAR constructed with anti-MSLN scFv and IL-15/IL-15Rα. **B** Flow cytometry plots demonstrating CAR expression on untransduced human T cells and CAR-T cells. UTD represented untransduced T cells. **C** Flow cytometry plots demonstrating IL-15/IL-15Rα on conventional CAR-T cells and CAR-IL-15/IL-15Rα-T cells. **D** Flow cytometry plots demonstrating the percentage of CAR^+^ and CD8.^+^ T cells in 15B6 and 15B6ss CAR-T after culture with gastric cancer cell line HGC27 for 24 h. Significant difference (*p < 0.05, **p < 0.01) between two groups was calculated using Independent-samples T test by IBM SPSS statistics 20

To assess the role of IL-15/IL-15Rα in CAR-T cells (CAR-ss-T), mesothelin (MSLN)-targeting CARs were constructed with or without IL-15Rα (Fig. 1A). Two different CARs were used to target MSLN. The receptor C4 targeting MSLN was a nanobody screened by VHH library and tested in CAR-T cells (Additional file 1: Figs. S1 and S2), while the receptor 15B6 was a single chain fragment of variation antibody [20]. Flow cytometry analysis showed comparable expression of CAR (Fig. 1B) and IL-15Rα (Fig. 1C) by C4, C4ss, 15B6 and 15B6ss CAR-T cells, indicating successful construction of CAR-ss-T cells. Besides, after co-culture with gastric cancer cell HGC27, the percentage of total CD8^+^ T cells and CAR^+^ T cell in CD8^+^ T cells was not changed significantly in CAR-ss-T (Fig. 1D), suggesting the existence of IL-15/IL-15Rα did not affect the proportion of CD8^+^ and CD4^+^ T cells.

**Fig. 2:**
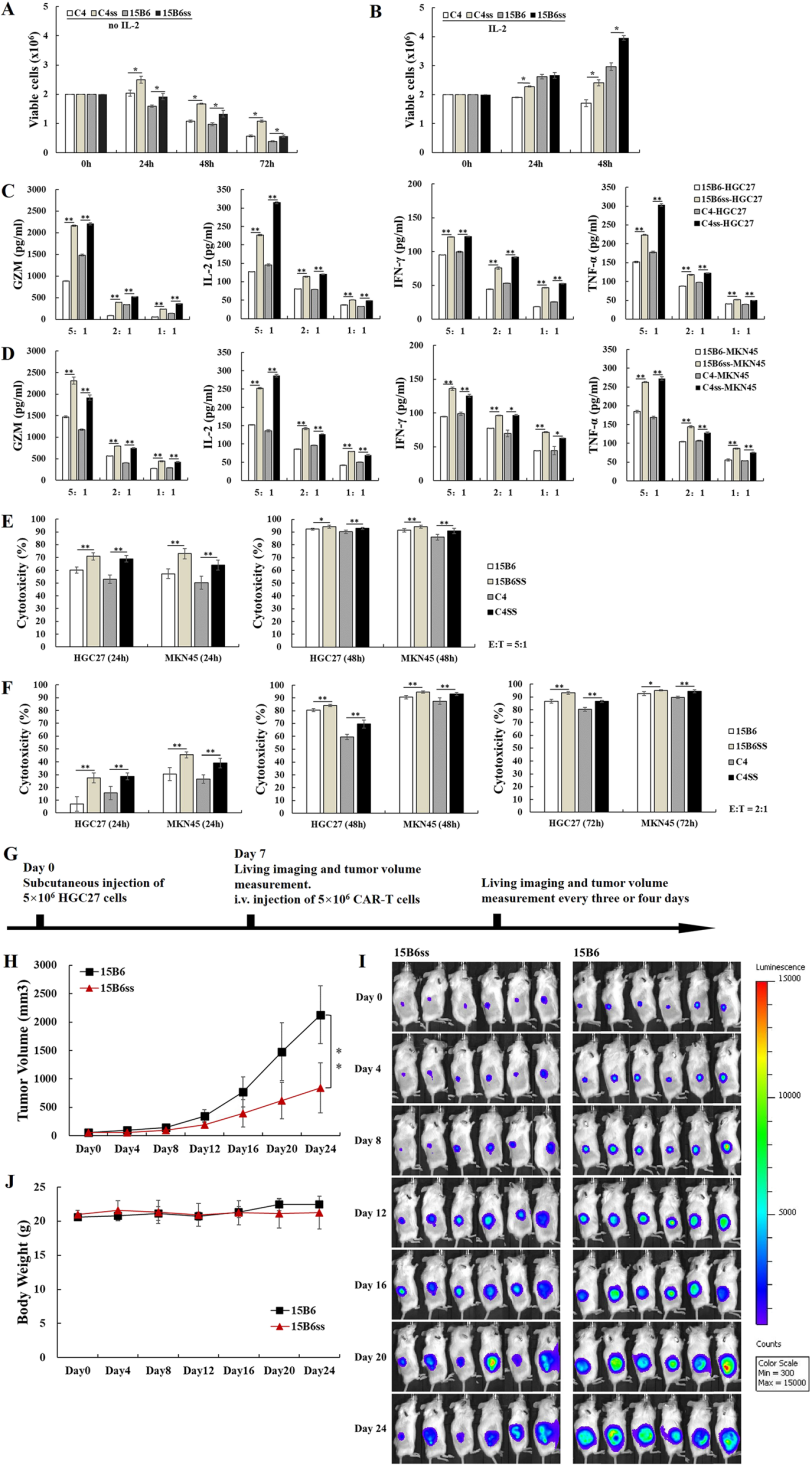
*In-vitro* cytotoxicity and cytokines production and *in-vivo* anti-tumor activity of CAR-IL15/IL-15Rα-T cells. Viable cells of CAR-T cells cultured without IL-2 (**A**) and with IL-2 (**B**). Cytokine production of CAR-T cells co-cultured with gastric cancer cell lines HGC27 (**C**) and MKN45 (**D**) under effector-to-target ratio of 5:1, 2:1 and 1:1 for 24 h by ELISA assay. **E** *In-vitro* cytotoxicity of CAR-T cells against HGC27 and MKN45 under effector-to-target ratio of 5:1 for 24 h and 48 h by bioluminescence assay. **F** *In-vitro* cytotoxicity of CAR-T cells against HGC27 and MKN45 under effector-to-target ratio of 2:1 for 24 h, 48 h and 72 h by bioluminescence assay. **G** Experiment setup for (**H**), (**I**) and (**J**). Tumor volume (**H**), bioluminescence imaging (**I**) and Mice body weight (**J**) of HGC27 mouse xenografts after treatment with 5 × 10^6^ 15B6 or 15B6ss CAR-T cells. Asterisks in figures represent significant difference (*p < 0.05, **p < 0.01) between two groups, calculated using Independent-samples T test by IBM SPSS statistics 20

To validate the function of engineered CAR-T cells and assess the impact of IL-15/IL-15Rα, proliferation, cytokine secretion, and tumor lytic capacity of CAR-T cells were examined. For cell proliferation, CAR-ss-T cells maintained more viable cells compared with conventional CAR-T in the absence of cytokine support (Fig. 2A). CAR-ss-T cells expanded faster than conventional CAR-T cells under interleukin-2 (IL-2) support (Fig. 2B). These results indicated that IL-15/IL-15Rα in CAR extracellular region enhanced cell viability and cell proliferation. Compared to conventional CAR-T cells, CAR-ss-T cells produced higher levels of interferon-γ (IFN-γ), IL-2, tumor necrosis factor-α (TNF-α) and granzyme B (GZM) against MSLN-expressing HGC27 and MKN45 tumor cells (Fig. 2C and D). Bioluminescence-based killing assay demonstrated stronger cytotoxicity of CAR-ss-T cells against HGC27 and MKN45 tumor cells (Fig. 2E and F). To determine the efficacy of CAR-ss-T cells in vivo, NOD-Prkdcscid IL2rgem1/Cyagen (C-NKG) mice were inoculated with 5 × 10^6^ HGC27 tumor cells subcutaneously, followed by intravenous injection of 15B6 CAR-T or 15B6ss CAR-T cells 7 day later (Fig. 2G). At a dose level of 5 × 10^6^ CAR-T cells, CAR-ss-T cells exhibited better tumor-control capability (Fig. 2H and I) without significant body weight change (Fig. 2J). Collectively, the data indicated that CAR-ss-T cells increased cell viability and cytokines production in vitro, and enhanced tumor control against gastric cancer compared with traditional CAR-T cells in vivo.

### GLIPR1 was up-regulated after CAR-T treatment and overexpressed in a proportion of stomach adenocarcinoma patients with decreased survival rate

**Fig. 3:**
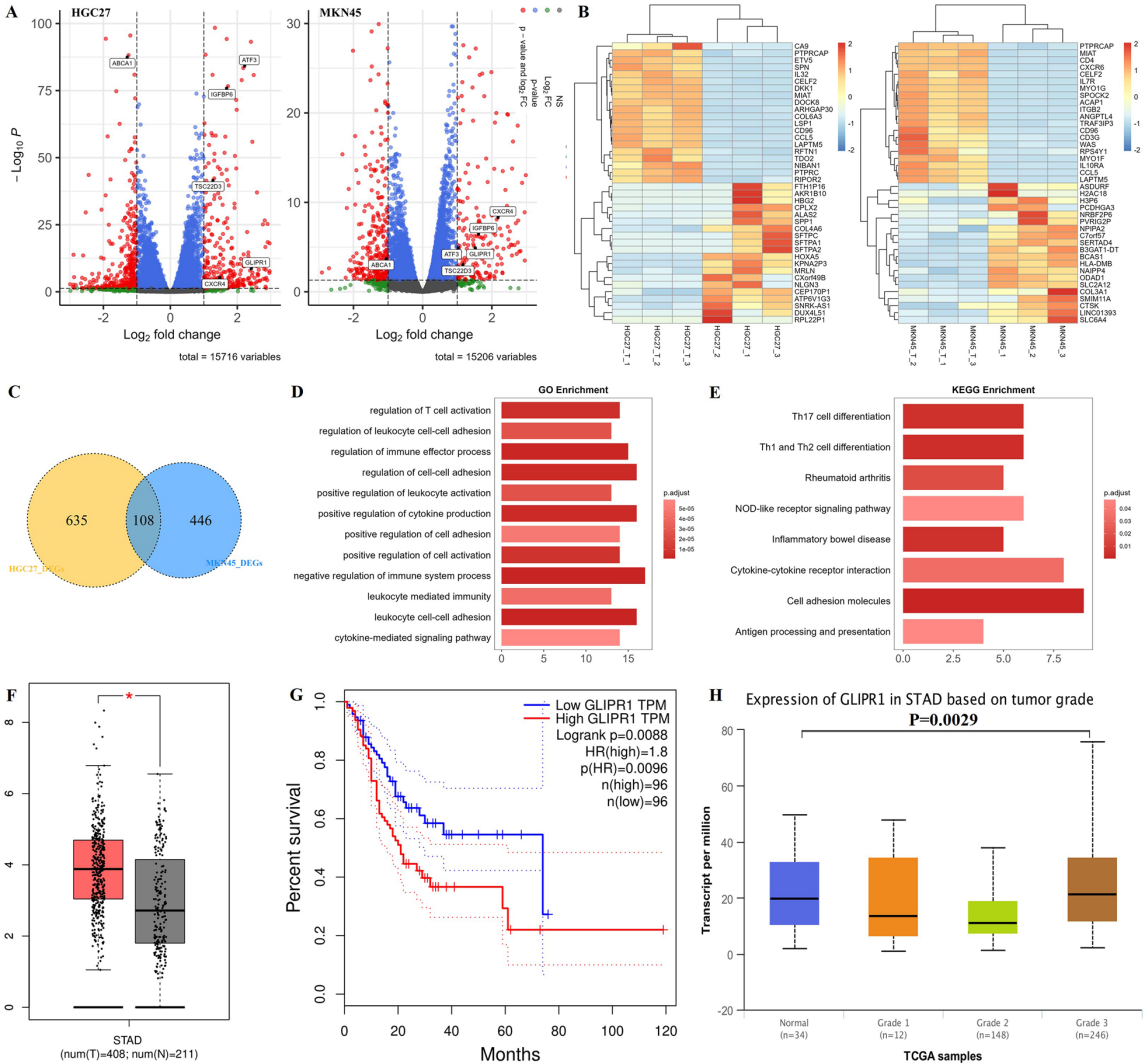
GLIPR1 was up-regulated after CAR-T treatment and over-expressed on parts of STAD patients. (**A**) Volcano plots of DEGs between control group (cancer cells only) and treatment group (co-culture of CAR-T cells and cancer cells). (**B**) Heat maps of top 20 most increased or decreased DEGs between control group (cancer cells only) and treatment group (co-culture of CAR-T cells and cancer cells). (**C**) Venn diagram of DEGs between two cancer cell lines HGC27 and MKN45. (**D**) Gene ontology analysis of overlapping DEGs between two cancer cell lines. (**E**) KEEG analysis of overlapping DEGs between two cancer cell lines. (**F**) The differential analysis of GLIPR1 between tumors and normal. The method for differential analysis is one-way ANOVA using disease state (Tumor or Normal) as variable for calculating differential expression. (**G**) Survival analysis of STAD patients between high-expression and low-expression cohorts based on quartile cutoff [21]. Significance of survival impact is measured by log ran test. (**H**) Gene expression of GLIPR1 in STAD patients based on grades [22]. Significant difference of gene expression is estimated by Student’s T test

To understand how tumor cells resist CAR-T cells and to find a more effective way CAR-T treatment like combined therapy, the conventional CAR-T (15B6 CAR-T) was co-cultured with HGC27 and MKN45 at a low effector-to-target ratio (1:1) for 24 h. Then the remaining tumor cells (co-culture group) and tumor cells cultured alone (control group) were RNA-sequenced and compared to identify differentially expressed genes (DEGs). The DEGs were displayed in volcano plots (Fig. 3A). There were 635 DEGs in HGC27 and 446 DEGs in MKN45 in the remaining tumor cells after CAR-T cell killing. The top 20 most increased or decreased DEGs were displayed in heat maps (Fig. 3B). To find out the most meaningful ones, DEGs from HGC27 and MKN45 were overlapped and 108 genes were found in common (Fig. 3C). Gene Ontology (GO) analysis of the mutual DEGs showed enriched biological processes, such as negative regulation of immune system process and positive regulation of cell adhesion (Fig. 3D). Kyoto Encyclopedia of Genes and Genomes (KEGG) analysis showed enriched pathways such as inflammatory bowel disease and antigen processing and presentation (Fig. 3E). Above all, our results displayed the real-time immune regulation of cancer cells against CAR-T cells.

To identify the DEGs that significantly affect the survival of stomach adenocarcinoma (STAD) patients, all DEGs were analyzed via Gene Expression Profiling Interactive Analysis 2 (GEPIA2) [21] and the University of ALabama at Birmingham CANcer data analysis Portal (UALCAN) [22]. There were 6 DEGs identified that significantly affect survival of STAD patients (Fig. 3A and Additional file 1: Fig. S3), including activating transcription factor 3 (ATF3), TSC22 domain family protein 3 (TSD22D3), insulin-like growth factor-binding protein 6 (IGFBP6), phospholipid-transporting ATPase 1 (ABCA1), C-X-C chemokine receptor type 4 (CXCR4) and GLI Pathogenesis Related 1 (GLIPR1). Given that membrane proteins were easier to target in therapy, GLIPR1 were chosen for further research, which was an oncogene or tumor-suppressed genes in different cancers, but barely understood in gastric cancer [23]. Based on The Cancer Genome Atlas Program (TCGA) and Genotype-Tissue Expression (GTEx) databases, GLIPR1 was overexpressed in some patients (Fig. 3F) and higher expression of GLIPR1 indicated worse survival (Fig. 3G), which was in consistent with its increasing in our results (Fig. 3A). Furthermore, expression of GLIPR1 was significantly higher in grade 3 STAD patients (Fig. 3H), suggesting its improvement on STAD development. The data above indicated GLIPR1 as a membrane protein is a promising target for cancer therapy.

### CAR-ss-T cell treatment was enhanced with GLIPR1 knockdown in gastric cancer cells

**Fig. 4:**
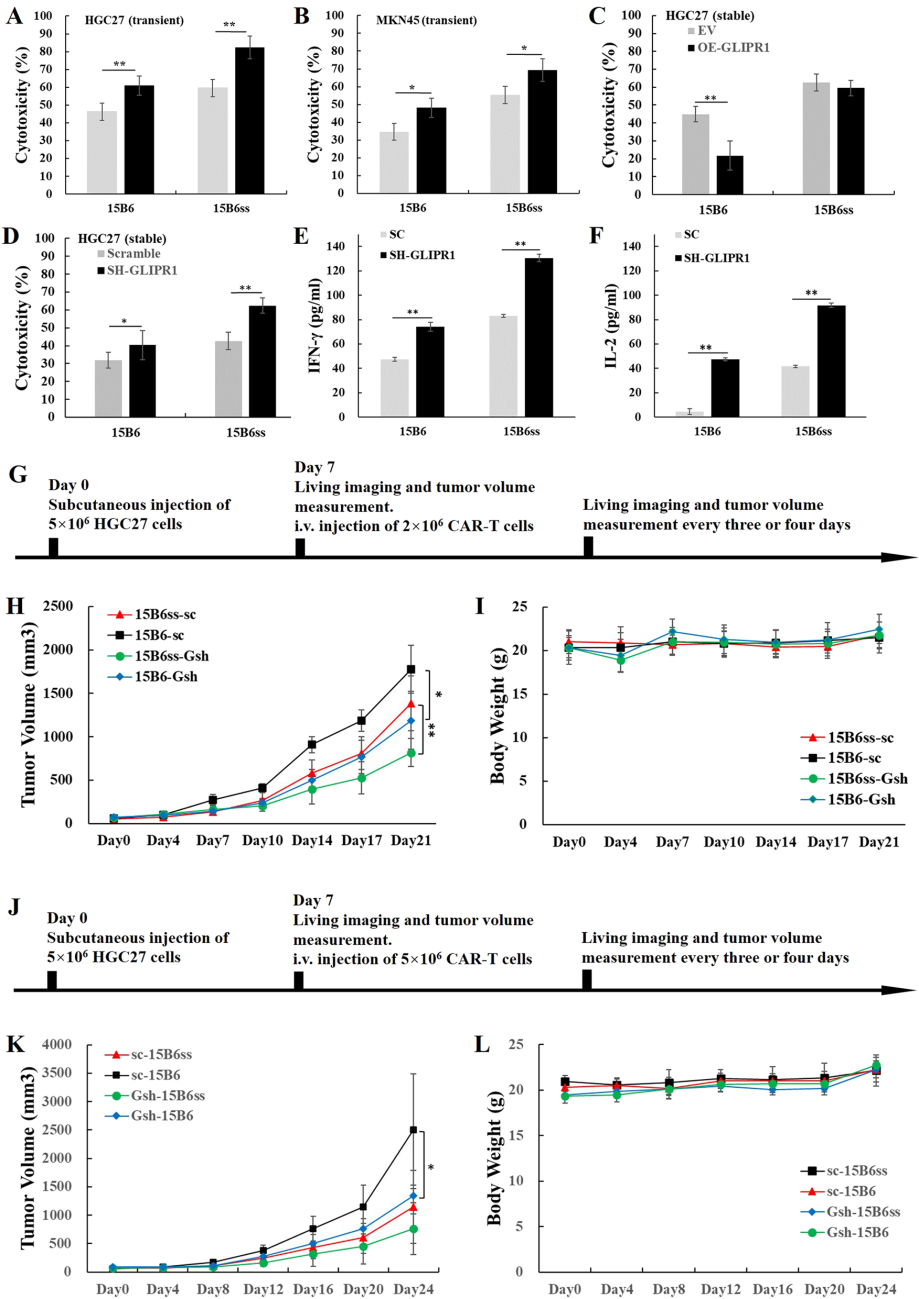
GLIPR1 knockdown enhanced CAR-T treatment in vitro and in vivo. **A** and **B** *In-vitro* cytotoxicity of 15B6 and 15B6ss CAR-T against gastric cancer cell lines HGC27 and MKN45 with GLIPR1 transient knockdown under effector-to-target ratio of 2:1 for 24 h by bioluminescence assay. **C** *In-vitro* cytotoxicity of 15B6 and 15B6ss CAR-T on HGC27 with GLIPR1 stable overexpression (OE-GLIPR1) under effector-to-target ratio of 2:1 for 24 h by bioluminescence assay. **D** *In-vitro* cytotoxicity of 15B6 and 15B6ss CAR-T on HGC27 with GLIPR1 stable knockdown (SH-GLIPR1) under effector-to-target ratio of 2:1 for 24 h by bioluminescence assay. **E** and **F** Cytokine production of CAR-T cells co-cultured with HGC27-Gsh under the effector-to-target ratio of 2:1 for 24 h by ELISA assay. (**G**) Experiment setup for (**H**) and (**I**). Tumor volume (**H**) and Mice body weight (**I**) of HGC27-sc and HGC27-Gsh mouse xenografts after treatment with 2 × 10^6^ 15B6 or 15B6ss CAR-T cells. Sc means scramble. Gsh means shRNA for GLIPR1. **J** Experiment setup for (**K)** and (**L**). Tumor volume (**K**) and Mice body weight (**L**) of HGC27-sc and HGC27-Gsh mouse xenografts after treatment with 5 × 10.^6^ 15B6 or 15B6ss CAR-T cells. Asterisks in figures represented significant difference (*p < 0.05, **p < 0.01) between two groups, calculated using one-way ANOVA test by IBM SPSS statistics 20

**Fig. 5:**
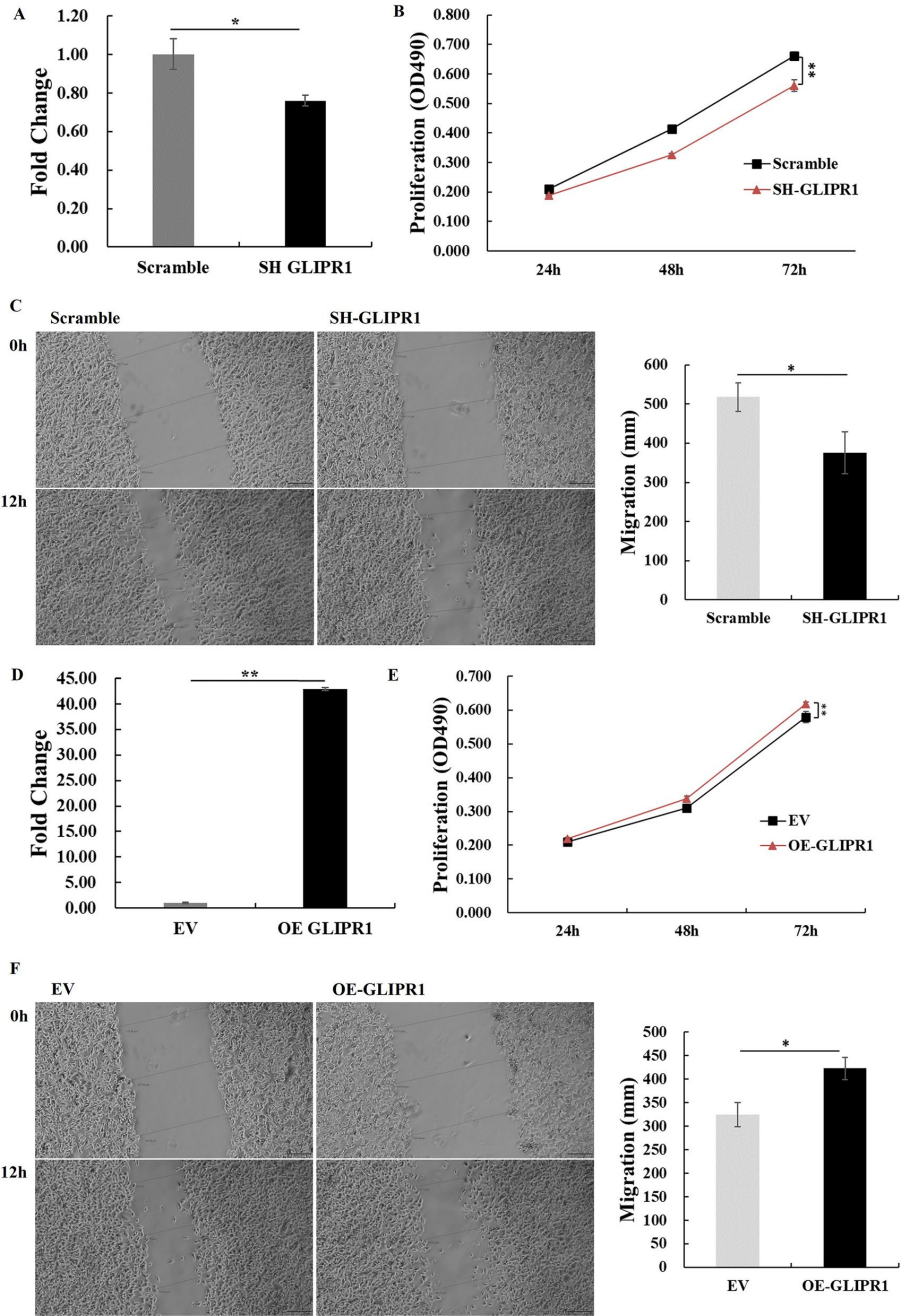
GLIPR1 knockdown enhanced inhibited gastric cancer cell proliferation and migration. **A** Down-regulation of GLIPR1 in HGC27 stable cell line was tested by qPCR. **B** Proliferation of HGC27 with GLIPR1 suppression was tested by CCK-8 assay. **C** Migration of HGC27-luc with GLIPR1 suppression was tested for 12 h by scratch assay. **D** GLIPR1 overexpression in HGC27 stable cell line was tested by qPCR. **E** Proliferation of HGC27 with GLIPR1 overexpression was tested by CCK-8 assay. **F** Migration of HGC27-luc with GLIPR1 overexpression was tested for 12 h by scratch assay. Asterisks in figures represented significant difference (*p < 0.05, **p < 0.01) between two groups, calculated using Independent-samples T test by IBM SPSS statistics 20

To validate the potential of GLIPR1 knockdown combined with CAR-T cells, the cytotoxicity and cytokine secretion of CAR-T cells were examined in response to gastric cancer cells with GLIPR1 knockdown. With transient down-regulation of GLIPR1 in HGC27 and MKN45, both conventional CAR-T and CAR-ss-T exhibited increased cytotoxicity (Fig. 4A and B). With stable overexpression of GLIPR1 in HGC27 (Fig. 5D), cytotoxicity of 15B6 CAR-T was inhibited while 15B6ss CAR-T was not affected, suggesting better tumor-killing capacity of CAR-ss-T cells. With stable suppression of GLIPR1 in HGC27 (Fig. 5A), both CAR-T cells increased cytotoxicity against cancer cells (Fig. 4D) and cytokine production of IFN-γ and IL-2 (Fig. 4E and F), suggesting CAR-T cells displayed better functions in gastric cancer with GLIPR1 knockdown. To determine the efficacy in vivo, C-NKG mice were inoculated subcutaneously with HGC27-Scramble (HGC27-sc) or HGC27-shRNA-GLIPR1 (HGC27-Gsh) tumor cells, followed by intravenous injection of 15B6 or 15B6ss CAR-T cells 7 day later (Fig. 4G). At a low dosage of 2 × 10^6^ CAR-T cells, both CAR-T cells exhibited better tumor-control capability (Fig. 4H) in HGC27-Gsh bearing mice without significant body weight change (Fig. 4I). At a higher dosage of 5 × 10^6^ CAR-T cells, tumors in HGC27-Gsh group were better controlled, though without complete remission (Fig. 4J–L). Collectively, GLIPR1 knockdown in gastric cancer cells enhanced the cytotoxicity and cytokine production of CAR-T cells in vitro and in vivo.

### GLIPR1 knockdown inhibited gastric cancer cell proliferation and migration

To better understand the effect of GLIPR1 knockdown in gastric cancer cells, cell proliferation, migration and invasion abilities were tested. With GLIPR1 down-regulation in HGC27 (Fig. 5A), tumor cell proliferation was inhibited (Fig. 5B). The scratch assay indicated the decreased migration of HGC27 with GLIPR1 down-regulation (Fig. 5C). In contrast, overexpression of GLIPR1 in HGC27 enhanced cell proliferation and migration (Fig. 5D–F). Collectively, GLIPR1 knockdown inhibited gastric cancer cell proliferation and migration, while GLIPR1 overexpression improved cancer development, suggesting a promising immunotherapy target in combined strategy.

## Discussion

In this study, we demonstrated that IL-15/IL-15Rα enhanced the function of CAR-T cells both quantitatively and qualitatively, evidenced by improved expansion and cytotoxicity in vitro, more robust production of IFN-γ, IL-2, TNF-α and granzyme B upon co-culture with cancer cells, and enhanced tumor control in vivo. Normally, IL-15Rα is expressed by T cells, NK cells, natural killer T cells, B cells, dendritic cells, monocytes, and macrophages [24, 25]. After binding to IL-15Rα, IL-15 is trans-presented to the IL-2R/IL-15Rβ and -γ heterodimer expressed on effector T, B, and NK cells, thereby enhancing survival and maturation of the effector cells for killing tumor cells [26, 27]. Moreover, IL-15/IL-15Rα complex promotes proliferation of naïve and memory phenotype peripheral CD4^+^ and CD8^+^ T Cells in vivo. Based on this mechanism, IL-15 superagonist has been developed and shown to significantly enhance NK cells and CD8^+^ T cells’ anti-tumor efficacy in cancer models [11]. It enhances the proliferation of human NK, CD4^+^ and CD8^+^T cells in humanized mice [28, 29], and exhibits potent immunostimulatory effects on NK, NKT and CD8^+^ T cells [30, 31]. It is demonstrated that CAR-T cells secreting IL-15/IL-15Ra complex display comparable efficacy as secreting IL-15 alone with reduced adverse effects in hematological cancer [32]. In this study, we constructed the IL-15Rα into the extracellular region of CAR structure, which enhanced the anti-tumor effects of CAR-T cells against gastric cancer compared with conventional CAR-T. The overexpression of GLIPR1 did not inhibit the cytotoxicity of CAR-ss-T significantly, suggesting the potential of this structure against tumor resistance.

Through phenotypic changes, cancer cells evade recognition and destruction by immune effector cells such as CTLs, which not only facilitates the development of cancer cells, but also promotes resistance to immunotherapies based on the cytotoxicity of CTLs, including checkpoint inhibitors and CAR-T cells. Clarifying these changes in real-time contact between cancer cells and immune cells can provide more promising targets for immune therapy and combined therapy. Our data showed a series of DEGs that changed under conventional CAR-T cells killing. Both GO and KEGG analysis indicated that immune effector cells and related immune activities were regulated, such as T cell activation, cytokine production and immune effector cell process. Among 6 genes that significantly decreased the survival rate of patients, TSD22D3 and IGFBP6 were up-regulated in our results but their expression in STAD patients was lower than in normal tissues, suggesting that CTLs exhibit cytotoxicity partially by down-regulating them in cancer cells. As for CXCR4 and GLIPR1, they were up-regulated in our results and in STAD patients, indicating their oncogenic effect on gastric cancer. These DEGs may be potential targets for immune therapy in gastric cancer.

Among the six DEGs identified above, CXCR4 and GLIPR1 are membrane proteins. Since CXCR4 were well-understood in gastrointestinal malignancies [33], we chose GLIPR1 for further research. Given that STAD patients with high expression of GLIPR1 showed worse survival, our results further demonstrated that GLIRP1 down-regulation by shRNA in gastric cancer increased cytotoxicity and cytokine production of both conventional CAR-T and CAR-ss-T cells in vitro and better tumor control in vivo, suggesting CAR-T cell therapy together with GLIRP1 knockdown of gastric cancer cells was a promising combination to increase efficacy. GLIPR1 is identified as an oncoprotein in some cancer types including gliomas, melanoma cancers, breast cancers, and Wilms tumors, but as a tumor suppressor in some other cancers, like bladder cancers, prostate cancers, thyroid cancers, and lung cancers [23]. As an oncogene, GLIPR1 is necessary for stemness of glioma stem cells [34]. GLIPR1 could promote migration and invasion of glioma [35] and epithelial-mesenchymal transition by mediating signal transducer and activator of transcription 3 (STAT3) pathway and IL-6 [36]. High expression of GLIPR1 increased proliferation of breast cancer [37] and invasion of melanoma [38]. In gastric cancer, we demonstrated for the first time that down-regulation of GLIPR1 inhibited cell proliferation and migration, while up-regulation of GLIPR1 did the opposite and inhibited the cytotoxicity of conventional CAR-T cell, which offered convincing evidence to target GLIPR1 in immunotherapy.

There are some limitations for this study. More cancer cell lines for cell line-derived xenografts or patient-derived xenografts could be used to test the efficacy of CAR-ss-T cells and combined therapy. In the follow-up study, antibody targeting GLIPR1 or adeno-associated virus targeting GLIPR1 gene could be developed for combined therapy with CAR-T cells. Furthermore, dual CARs could be constructed that are bispecific for GLIPR1 and another target.

## Conclusions

CAR-T cells with an IL-15/IL-15Rα sushi domain displayed enhanced anti-tumor efficacy compared with conventional CAR-T, and GLIRP1 knockdown in gastric cancer further promoted the function of CAR-T cells. Our data provides a novel target for immunotherapy and a potential combined strategy for gastric cancer.

### Supplementary Information

**Additional file 1: Figure S1.** Competent CAR construction and cytotoxicity confirmation. (A) The workflow of phage-display screening for anti-MSLN VHH. (B) MSLN peptides for phage-display screening and CAR structure for the following construction. The peptide MSLN-1 (3CL) is a region of MSLN away from membrane and the MSLN-2 (2VV) is a juxtamembrane region. (C) CAR-Jurkat conducted using CARs derived from 29 VHH antibodies previously determined to specifically bind MSLN. Jurkat represented human T cell line. LNCap represented MSLN-negative cell line, HGC27 represented MSLN-positive cell line. (D) Flow cytometry plots demonstrating CAR expression on human T cell line Hut78. (E) In-vitro cytotoxicity of CAR-T cells against HGC27 under the effector-to-target ratio of 5:1 for 24 h by bioluminescence assay. (F) Flow cytometry plots demonstrating CAR expression on 3 healthy donors #2, #7 and #12. UTD represented untransduced T cells. Mock represented T cells transduced with no VHH CAR. (G) In-vitro cytotoxicity of CAR-T cells from 3 donors against HGC27 under the effector-to-target ratio of 10:1, 5:1 and 1:1 for 24 h by bioluminescence assay. Asterisks in figures represented significant difference (*p < 0.05, **p<0.01) between two groups, calculated using Independent-samples T test by IBM SPSS statistics 20. **Figure S2.** In-vitro cytotoxicity and in-vivo anti-tumor activity of C4-CAR-T. (A) Flow cytometry plots demonstrating MSLN expression on gastric cancer cell lines HGC27 and MKN45, pancreatic cell line ASPC-1 and squamous cell lung carcinoma cell line NCI-H520. (B) In-vitro cytotoxicity of C4 CAR-T on HGC27, ASPC1 and NCI-H520 for 24 h by bioluminescence assay. Bioluminescence imaging (C) and tumor volume (D) of HGC-27 mouse xenografts after treatment with 5×10^6^ untransduced T cell and C4 CAR-T cells. Asterisks in figures represented significant difference (*p < 0.05, **p<0.01) between two groups, calculated using Independent-samples T test by IBM SPSS statistics 20. **Figure S3.** DEGs that significantly affect survival and their expression difference between patients and normal tissues. (A) The differential analysis of 6 DEGs between tumors and normal. The method for differential analysis is one-way ANOVA, using disease state (Tumor or Normal) as variable for calculating differential expression. Red represented tumor. Grey represented normal. (B) Survival analysis of STAD patients between high-expression and low-expression cohorts of 6 DEGs based on quartile cutoff (21). Significance of survival impact is measured by log ran test.

## Data Availability

The data that support the findings of this study are available on request from the corresponding author, Y.L.

## Abbreviations

**CAR**: Chimeric antigen receptor
**GLIPR1**: GLI Pathogenesis Related 1
**STAD**: Stomach adenocarcinoma
**NK cells**: Natural killer cells
**CTLs**: Cytotoxic T lymphocytes
**CAR-ss-T**: CAR-IL-15/IL-15Rα-T
**IL-2**: Interleukin-2
**IFN-γ**: Interferon-γ
**TNF-α**: Tumor necrosis factor-α
**GZM**: Granzyme B
**C-NKG mice**: NOD-Prkdcscid IL2rgem1/Cyagen mice
**DEGs**: Differentially expressed genes
**GO**: Gene Ontology
**KEGG**: Kyoto encyclopedia of genes and genomes
**GEPIA2**: Gene expression profiling interactive analysis 2
**UALCAN**: The University of ALabama at Birmingham CANcer data analysis Portal
**ATF3**: Activating transcription factor 3
**TSD22D3**: TSC22 domain family protein 3
**IGFBP6**: Insulin-like growth factor-binding protein 6
**ABCA1**: Phospholipid-transporting ATPase 1
**CXCR4**: C-X-C chemokine receptor type 4
**TCGA**: The cancer genome atlas program
**GTEx**: Genotype-tissue expression databases
