# Supplementary material for: Combined therapy of CAR-IL-15/IL-15Rα-T cells and GLIPR1 knockdown in cancer cells enhanced anti-tumor effect against gastric cancer
Source: J Transl Med. 2024 Feb 18;22:171. doi: 10.1186/s12967-024-04982-6 (PMC10874561; doi:10.1186/s12967-024-04982-6)
Supplement: Supplementary file 1 — Additional file 1: Figure S1. Competent CAR construction and cytotoxicity confirmation. (A) The workflow of phage-display screening for anti-MSLN VHH. (B) MSLN peptides for phage-display screening and CAR structure for the following construction. The peptide MSLN-1 (3CL) is a region of MSLN away from membrane and the MSLN-2 (2VV) is a juxtamembrane region. (C) CAR-Jurkat conducted using CARs derived from 29 VHH antibodies previously determined to specifically bind MSLN. Jurkat represented human T cell line. LNCap represented MSLN-negative cell line, HGC27 represented MSLN-positive cell line. (D) Flow cytometry plots demonstrating CAR expression on human T cell line Hut78. (E) In-vitro cytotoxicity of CAR-T cells against HGC27 under the effector-to-target ratio of 5:1 for 24 h by bioluminescence assay. (F) Flow cytometry plots demonstrating CAR expression on 3 healthy donors #2, #7 and #12. UTD represented untransduced T cells. Mock represented T cells transduced with no VHH CAR. (G) In-vitro cytotoxicity of CAR-T cells from 3 donors against HGC27 under the effector-to-target ratio of 10:1, 5:1 and 1:1 for 24 h by bioluminescence assay. Asterisks in figures represented significant difference (*p < 0.05, **p<0.01) between two groups, calculated using Independent-samples T test by IBM SPSS statistics 20. Figure S2. In-vitro cytotoxicity and in-vivo anti-tumor activity of C4-CAR-T. (A) Flow cytometry plots demonstrating MSLN expression on gastric cancer cell lines HGC27 and MKN45, pancreatic cell line ASPC-1 and squamous cell lung carcinoma cell line NCI-H520. (B) In-vitro cytotoxicity of C4 CAR-T on HGC27, ASPC1 and NCI-H520 for 24 h by bioluminescence assay. Bioluminescence imaging (C) and tumor volume (D) of HGC-27 mouse xenografts after treatment with 5×10^6^ untransduced T cell and C4 CAR-T cells. Asterisks in figures represented significant difference (*p < 0.05, **p<0.01) between two groups, calculated using Independent-samples T test by IBM SPSS statistics 20. Fig [file 12967_2024_4982_MOESM1_ESM.docx]

**Supplementary Figures**


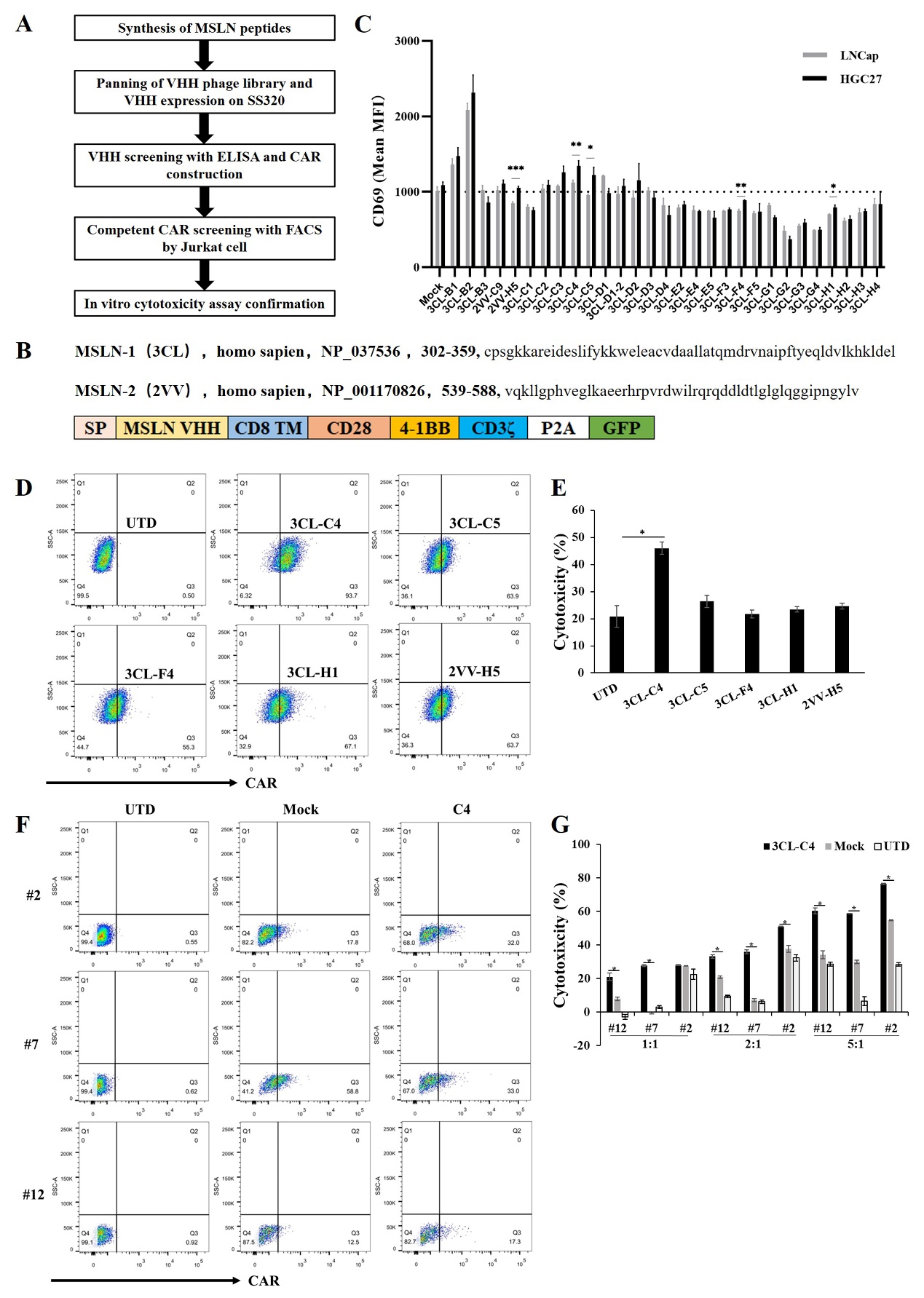


**Fig.S1 Competent CAR construction and cytotoxicity confirmation.**

(A) The workflow of phage-display screening for anti-MSLN VHH. (B) MSLN peptides for phage-display screening and CAR structure for the following construction. The peptide MSLN-1 (3CL) is a region of MSLN away from membrane and the MSLN-2 (2VV) is a juxtamembrane region. (C) CAR-Jurkat conducted using CARs derived from 29 VHH antibodies previously determined to specifically bind MSLN. Jurkat represented human T cell line. LNCap represented MSLN-negative cell line, HGC27 represented MSLN-positive cell line. (D) Flow cytometry plots demonstrating CAR expression on human T cell line Hut78. (E) In-vitro cytotoxicity of CAR-T cells against HGC27 under the effector-to-target ratio of 5:1 for 24 h by bioluminescence assay. (F) Flow cytometry plots demonstrating CAR expression on 3 healthy donors #2, #7 and #12. UTD represented untransduced T cells. Mock represented T cells transduced with no VHH CAR. (G) In-vitro cytotoxicity of CAR-T cells from 3 donors against HGC27 under the effector-to-target ratio of 10:1, 5:1 and 1:1 for 24 h by bioluminescence assay. Asterisks in figures represented significant difference (**p* < 0.05, ***p*<0.01) between two groups, calculated using Independent-samples T test by IBM SPSS statistics 20.


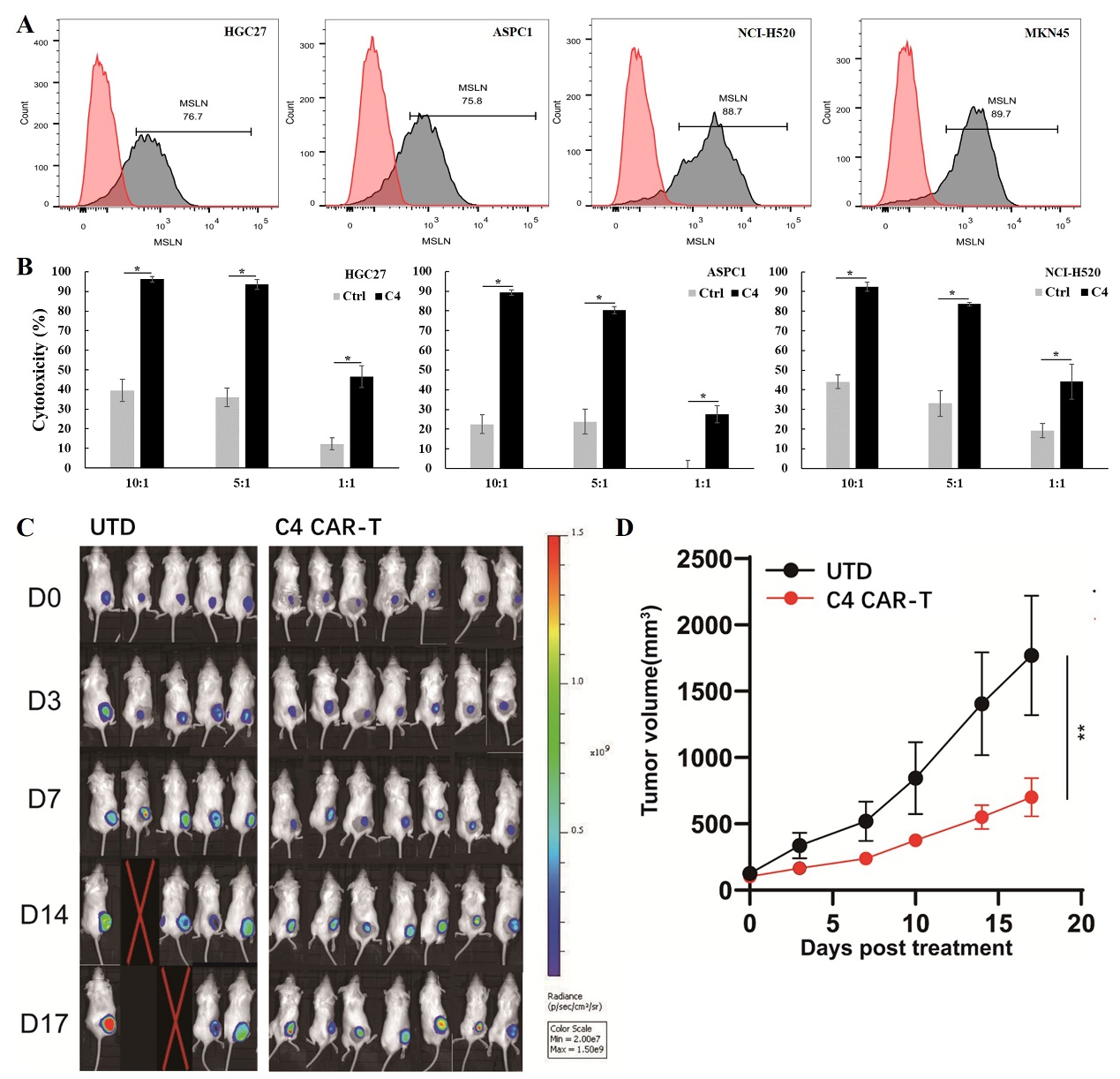


**Fig.S2 *In-vitro* cytotoxicity and in-vivo anti-tumor activity of C4 CAR-T**

(A) Flow cytometry plots demonstrating MSLN expression on gastric cancer cell lines HGC27 and MKN45, pancreatic cell line ASPC-1 and squamous cell lung carcinoma cell line NCI-H520. (B) In-vitro cytotoxicity of C4 CAR-T on HGC27, ASPC1 and NCI-H520 for 24 h by bioluminescence assay. Bioluminescence imaging (C) and tumor volume (D) of HGC-27 mouse xenografts after treatment with 5×10^6^ untransduced T cell and C4 CAR-T cells. Asterisks in figures represented significant difference (**p* < 0.05, ***p*<0.01) between two groups, calculated using Independent-samples T test by IBM SPSS statistics 20.


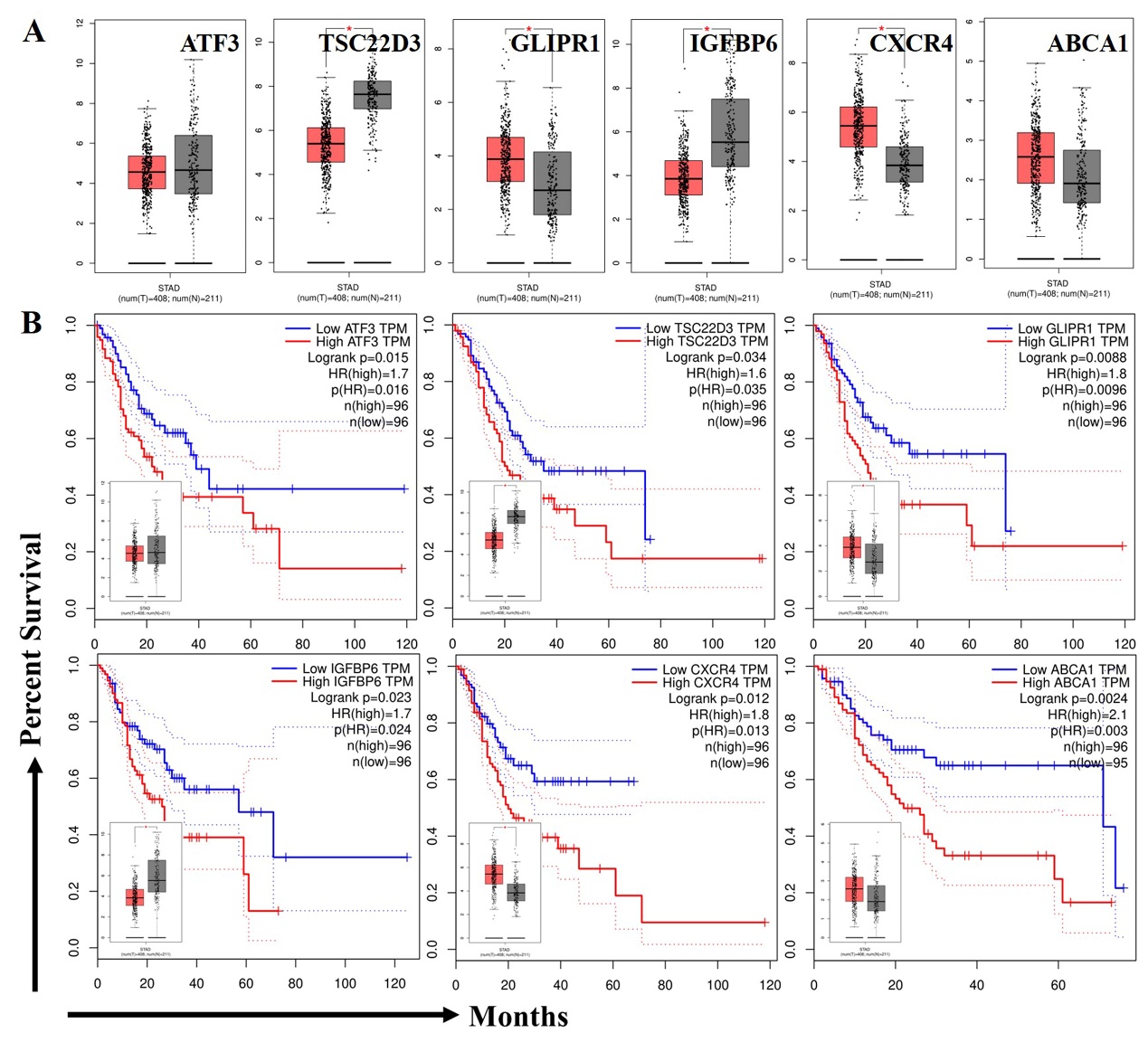


**Fig.S3 DEGs that significantly affect survival and their expression difference between patients and normal tissues**

(A) The differential analysis of 6 DEGs between tumors and normal. The method for differential analysis is one-way ANOVA, using disease state (Tumor or Normal) as variable for calculating differential expression. Red represented tumor. Grey represented normal. (B) Survival analysis of STAD patients between high-expression and low-expression cohorts of 6 DEGs based on quartile cutoff [1]. Significance of survival impact is measured by log ran test.

1. Tang, Z., et al., *GEPIA2: an enhanced web server for large-scale expression profiling and interactive analysis.* Nucleic Acids Res, 2019. **47**(W1): p. W556-w560.
